# SOX9-PDK1 axis is essential for glioma stem cell self-renewal and temozolomide resistance

**DOI:** 10.18632/oncotarget.22773

**Published:** 2017-11-30

**Authors:** Zhen Wang, Xiaoshan Xu, Nan Liu, Yingduan Cheng, Weilin Jin, Pengxing Zhang, Xin Wang, Hongwei Yang, Hui Liu, Yanyang Tu

**Affiliations:** ^1^ Department of Experimental Surgery, Tangdu Hospital, Fourth Military Medical, University, Xi’an 710038, China; ^2^ Department of Research, Cipher Ground, North Brunswick, NJ 08902, USA; ^3^ Institute of Nano Biomedicine and Engineering, Department of Instrument Science and Engineering, Key Laboratory for Thin Film and Microfabrication Technology of Ministry of Education, School of Electronic Information and Electronic Engineering, Shanghai Jiao Tong University, Shanghai 200240, China; ^4^ Department of Neurosurgery, Brigham and Women’s Hospital, Harvard Medical School, Boston, MA 02115, USA; ^5^ Center for Mitochondrial Biology and Medicine, The Key Laboratory of Biomedical Information Engineering of Ministry of Education, School of Life Science and Technology and Frontier Institute of Science and Technology, Xi’an Jiao Tong University, Xi’an 710049, China

**Keywords:** glioblastoma multiforme, SOX9, PDK1, self-renewal, temozolomide

## Abstract

Glioblastoma multiforme (GBM) is the most common and aggressive brain tumor with limited therapeutic options. Glioma stem cell (GSC) is thought to be greatly responsible for glioma tumor progression and drug resistance. But the molecular mechanisms of GSC deriving recurrence and drug resistance are still unclear. *SOX9* (sex-determining region Y (SRY)-box9 protein), a transcription factor expressed in most solid tumors, is reported as a key regulator involved in maintaining cancer hallmarks including the GSCs state. Previously, we have observed that silencing of SOX9 suppressed glioma cells proliferation both *in vitro* and *in vivo*. Here, we found that SOX9 was essential for GSC self-renewal. Silencing of SOX9 down-regulated a broad range of stem cell markers and inhibited glioma cell colony and sphere formation. We identified pyruvate dehydrogenase kinase 1 (*PDK1*) as a target gene of SOX9 using microarray analyses. PDK1 inactivation greatly inhibited glioma cell colony and sphere formation and sensitized glioma spheres to temozolomide (TMZ) toxicity. In addition, SOX9-shRNA and PDK1 inhibitor could greatly sensitize GSC to TMZ *in viv*o. Taken together, our data reveals that SOX9-PDK1 axis is a key regulator of GSC self-renewal and GSC temozolomide resistance. These findings may provide help for future human GBM therapy.

## INTRODUCTION

Glioblastoma multiforme (GBM) is regarded as one of the most common intracranial primary tumors. For years, conventional therapeutic strategies including surgery, radiation and chemotherapy targeting the bulk of tumor have acquired limited benefits, with the median survival hardly improved [1]. Recurrence of malignant tumor is one of the leading causes of cancer patients’ death and one of the biggest challenges in cancer treatment currently. Although biotechnology helps people understand cancer more than before, the primary cause of tumorigenesis, recurrence is still unclear. As the culprit who accounts for the relapse and therapeutic resistance, the small group called “glioma stem-like cell” (GSC) demonstrates many features as neural stem cells like sphere formation [2–5], self-renewal and differentiation and has been thought to be the potential cause of tumor recurrence during glioma cancer therapy [6]. Hence, specific targeted therapies aiming at elimination of GSC would render great significance to therapeutic advancement of GBM.

Sex-determining region Y (SRY)-box9 protein (*SOX9*) is a transcription factor that controls cell fate decision during the development and homeostasis of a broad range of tissues, and is expressed in a wide range of cancers [7–10]. SOX9 deletion prevents tumorigenesis in prostatic and pancreatic mouse cancer models [11, 12]. Overexpression of SOX9 inhibits apoptosis and promotes proliferation, invasion, and migration, whereas down-regulation of SOX9 impaired invasion and growth *in vitro* and *in vivo* [13–15]. SOX9 has been well recognized for its capacity in stemness maintenance of neural stem cell (NSC). Gain- and loss-of-function studies indicated that SOX9 was essential for multipotent NSC formation. Moreover, sonic Hedgehog was able to stimulate precocious generation of NSCs by inducing SOX9 expression [16]. SOX9 has been also well characterized for its oncogenic potency in many aspects of cancer stemness [9], such as promoting tumor initiation and invasion, maintaining the self-renewal of CSCs [17]. However, the mechanism for aberrant up-regulation of SOX9 in GBM remains elusive.

In this article, we found that SOX9 mRNA was overexpressed and positively correlated with the protein level in GBM. Knockdown of SOX9 in GBM cell lines markedly suppressed the stem cell-like properties, including stem cell marker expression level and glioma cell sphere formation, indicating that SOX9 was essential for GSC self-renewal. We also found that pyruvate dehydrogenase kinase 1 (*PDK1*) was a downstream target of SOX9 through microarray analyses, and the activity of PDK1 was essential for GSC self-renewal in GBM. Inactivation of PDK1 greatly inhibited glioma cell sphere formation. In addition, PDK1 inactivation greatly sensitized glioma sphere to temozolomide. The level of phosphorylation AKT was regulated by PDK1 activity, which was important for GSC temozolomide resistance. Collectively, these results indicated that SOX9-PDK1 axis was a critical regulator of GSC self-renewal and played important role in GBM drug resistance.

## RESULTS

### SOX9 was overexpressed both at the mRNA level and protein level in GBM

**Figure 1:**
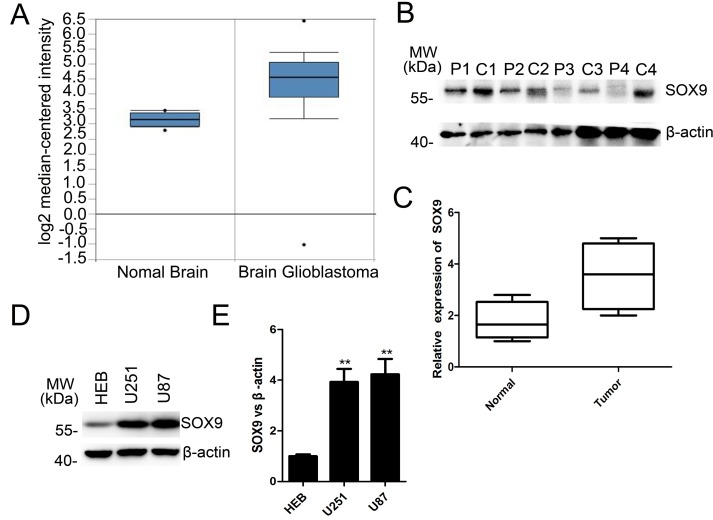
SOX9 was overexpressed both at the mRNA level and protein level in GBM (**A**) Relative mRNA expression of SOX9 in normal brain and GBM samples TCGA database. (**B**) Western blots analysis for protein expression of SOX9 in patients’ tissue samples. P represented for normal tissue, C represented for GBM tissue. (**C**) Box plots of SOX9 expression in tissue samples. (**D**) Western blots analysis for protein expression of SOX9 in U251, U87 and HEB cell lines. (**E**) Quantitation of SOX9 expression in U251, U87 and HEB cell lines. The densitometry data were expressed as the mean±SD of three independent experiments. ^**^P < 0.01, and ^***^P < 0.001 versus relative control.

To investigate the candidate role of SOX9 in GBM, we firstly interrogated the data of SOX9 by bioinformatics using TCGA database and Human protein Atlas. We found that the mRNA level of SOX9 was markedly higher in GBM tissues compared with normal brain from the 542 GBM samples in TCGA database (http://tcga-data.nci.nih.gov) (Figure 1A). The protein levels of SOX9 were majority high in most GBM sample in Human protein Atlas (http://www.proteinatlas.org). Nest, the protein expression of SOX9 was detected in four GBM tissues and the couple normal brain tissues. Western blotting analyzing verified that SOX9 expressed significant higher in GBM tissues than normal brain tissues (Figure 1B and 1C).Then, the SOX9 expression level was also detected in U251 and U87 GBM cell lines and HEB, and the result showed that the level of SOX9 was higher in both U251 cells and U87 cells than in HEB cells (Figure 1D and 1E).On the whole, these data reflected that SOX9 was overexpressed in human GBM.

### SOX9 was at high expression in glioma stem cell-like sphere

**Figure 2:**
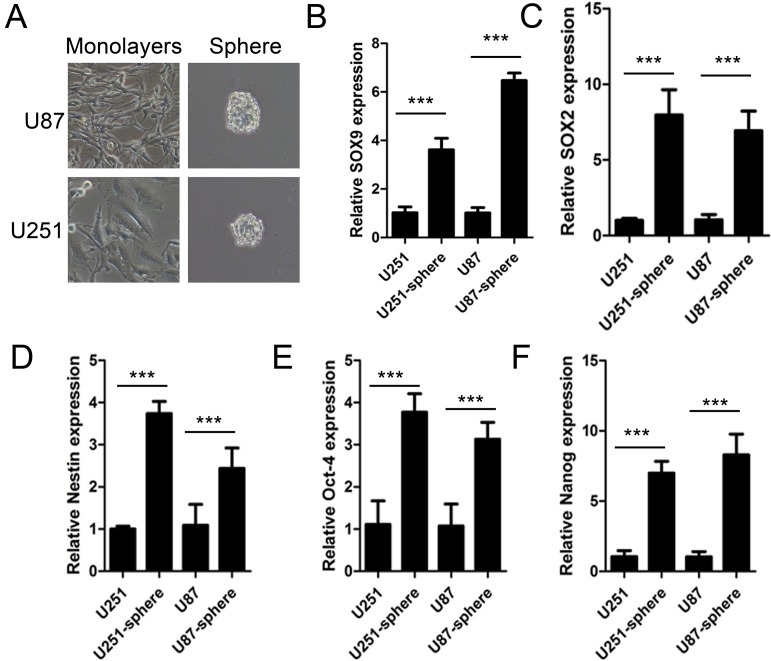
SOX9 was at high expression in glioma stem cell-like sphere (**A**) Representative images for spheres of U251MG and U87MG cells. Magnification, ×200. (**B**–**F**) qRT-PCR detection for expression of SOX9, SOX2, NESTIN, Oct-4, NANOG, respectively. The densitometry data were expressed as the mean ± SD of three independent experiments. ^***^P < 0.001 versus normal U87 or U251 cells.

Stem cell-like sphere was a common model frequently used in stem cell studies. To verify the potential role of SOX9 in glioma stem cells, sphere formation assay was processed using U251MG and U87MG cell lines (Figure 2A). Data showed that mRNA level of SOX9 was obviously higher in spheres than in monolayers (Figure 2B). Simultaneously, levels of several stem cell makers SOX2 (Figure 2C), NESTIN (Figure 2D), Oct-4 (Figure 2E) and NANOG (Figure 2F), were all higher compared with in monolayers. These results indicated that the level of SOX9 and stem cell marker increased in glioma spheres.

### SOX9 knockdown inhibits glioma cell colony formation and stem cell-like properties

**Figure 3:**
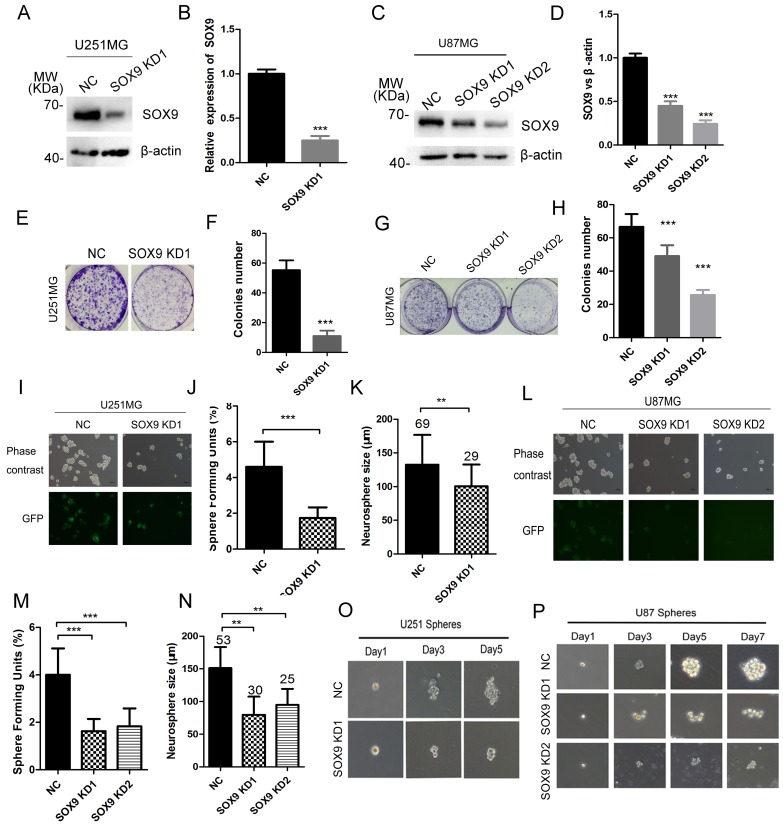
SOX9 knockdown inhibited glioma cell colony formation and sphere formation (**A**) The efficiency validation of SOX9-shRNAs by immunoblotting in U251 cells. (**B**) Quantitation of SOX9 expression in SOX9 knockdown and control U251 cells. (**C**) The efficiency validation of SOX9-shRNAs by immunoblotting in U87 cells. (**D**) Quantitation of SOX9 expression in SOX9 knockdown and control U87 cells. (**E**) The morphology of cell colonies formed by SOX9 knockdown U251 cells. (**F**) Number of cell colonies formed by SOX9 knockdown and control U251 cells. (**G**) The morphology of cell colonies formed by SOX9 knockdown U87 cells. (**H**) Number of cell colonies formed by SOX9 knockdown and control U87 cells. (**I**) Represent images of U251-SOX9 knockdown spheres. (**J**) Effect of SOX9 knockdown on the number of Sphere Forming Units (SFU) of U251 cells. (**K**) Diameters of glioma spheres decreased in SOX9 knockdown U251 spheres, Arabic numerals represented for spheres number. (**L**) Represent images of U87-SOX9 knockdown spheres. (**M**) Effect of SOX9 knockdown on the number of SFU of U87 cells. (**N**) Diameters of glioma spheres decreased in SOX9 knockdown U87 spheres, Arabic numerals represented for spheres number. (**O**) Single sphere growth in SOX9 knockdown U251MG. (**P**) Single sphere growth in SOX9 knockdown U87MG. Data were expressed as the mean ± SD of three independent experiments. Figures presented are the representative of at least three independent experiments. ^**^P < 0.01, and ^***^P < 0.001 versus NC.

To determine the biological function of SOX9 in glioma stemness, we applied shRNAs against SOX9 to U251 and U87MG cells, and found that SOX9-shRNAs significantly decreased SOX9 protein expression in U251 (Figure 3A and 3B) and U87MG cells (Figure 3C and 3D). The stable cell lines constructed by SOX9-shRNAs lentivirus (LV3-GFP) were subsequently applied in the colony formation and sphere formation assay. Stably expressing SOX9 shRNA significantly decreased the colony formation ability both in U251MG (Figure 3E and 3F) and U87MG (Figure 3G and 3H) compared to those cell lines stably expressing negative control shRNA. The sphere-forming units (SFU) and diameter of spheres were detected to access the effect of SOX9 knockdown on glioma sphere formation. Result showed that SOX9 knockdown significantly decreased the SFU and diameter of spheres in U251MG (Figure 3I, 3J and 3K) and U87MG (Figure 3L, 3M and 3N) compared to their negative controls. Moreover, clone-forming ability at a single cell level was markedly inhibited by treatment with SOX9 shRNA compared with the control shRNA both in U251 (Figure 3O) and in U87 cells (Figure 3P). These founding demonstrated that SOX9 was essential for glioma cell stemness.

**Figure 4:**
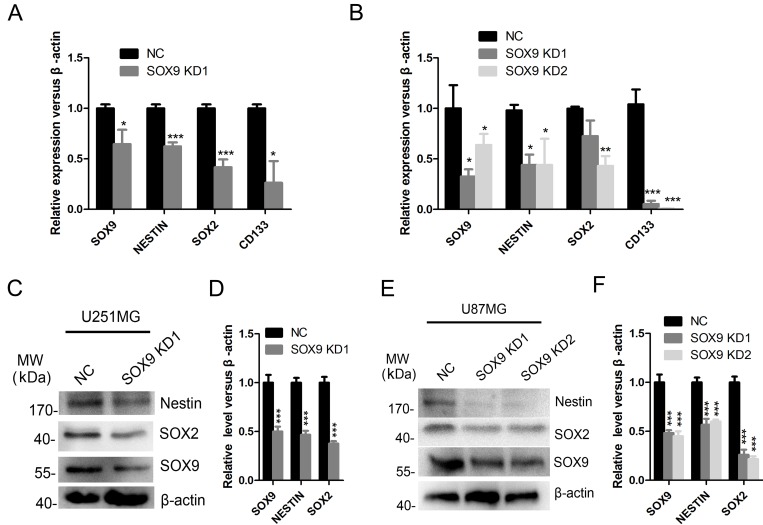
SOX9 regulated the expression of stem cell markers in glioma cells (**A**) qRT-PCR detection for expression of SOX9, SOX2, NESTIN, CD133 in U251MG. (**B**) qRT-PCR detection for expression of SOX9, SOX2, NESTIN, CD133 in U87MG. (**C**) Western blot analysis for expression of SOX9, SOX2 and NESTIN in SOX9 knockdown and control U251 cells. (**D**) Quantitation of the expression of SOX9, SOX2 and NESTIN in SOX9 knockdown and control U251 cells. (**E**) Western blot analysis for expression of SOX9, SOX2 and NESTIN in U87 cells. (**F**) Quantitation of the expression of SOX9, SOX2 and NESTIN in SOX9 knockdown and control U87 cells. The densitometry data were expressed as the mean ± SD of three independent experiments. ^*^P < 0.05, ^**^P < 0.01, ^***^P < 0.001 versus NC.

In addition, knockdown of SOX9 in U251MG and U87MG cell lines decreased the mRNA level of stem cell markers CD133, NESTIN and SOX2 in both U251MG (Figure 4A) and U87MG (Figure 4B) cell lines. Protein level of NESTIN and SOX2 was also decreased in both U251MG (Figure 4C and 4D) and U87MG (Figure 4E and 4F) cell lines after SOX9 was silenced. Taken together, the above results indicated that SOX9 was a putative cancer stem marker in GBM cells and was essential for glioma stem cell properties.

### PDK1 is a downstream target of SOX9

**Figure 5:**
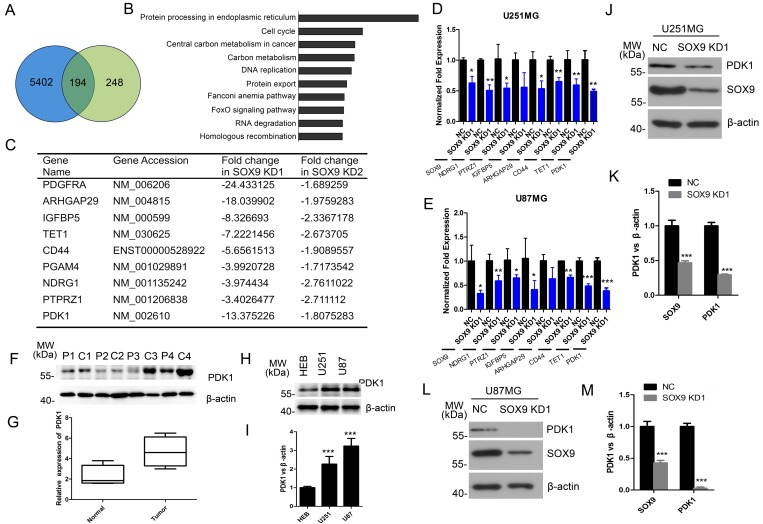
PDK1 is a downstream target of SOX9 **(A)** Venn diagram showing the intersection of genes between: genes that were downregulated and upregulated 1.5-fold or more in SOX9 knockdown U251 cells. (**B)** Pathway enrichment analysis for 506 genes identified from SOX9-shRNA1 and SOX9-shRNA2. (**C)** Representative genes that were found to be downregulated in Microarray analyses. (**D)** qRT-PCR validation of 8 mutually regulated gene in knockdown U251 cells. (**E)** qRT-PCR validation of 8 mutually regulated gene in knockdown U251 cells. (**F)** Western blots analysis for protein expression of PDK1 in patients’ tissue samples. P represented for normal tissue, C represented for GBM tissue. (**G)** Box plots of PDK1 expression in tissue samples. (**H)** Western blots analysis for protein expression of PDK1 in U251, U87 and HEB cell lines. (**I)** Quantitation of PDK1 expression in U251, U87 and HEB cell lines. (**J)** Western blot analysis verified the expression of PDK1 in SOX9 knockdown and control U251 cells. (**K)** Quantitation of the expression of PDK1 in SOX9 knockdown and control U251 cells. (**L)** Weatern blot analysis verified the expression change of PDK1 in SOX9 knockdown U87 cells. (**M)** Quantitation of the expression of PDK1 in SOX9 knockdown and control U87 cells.The densitometry data were expressed as the mean ± SD of three independent experiments. ^*^P < 0.05, ^**^P < 0.01, ^***^P < 0.001 versus NC.

Given that the SOX9 was involved in glioma stem cell-like properties, we were interested in the relevant downstream genes regulated by SOX9 in glioma. There were a total of about 194 genes with at least a 1.5-fold change mutually regulated by SOX9 in U251 cells by both two SOX9-shRNA1 and SOX9-shRNA2 (Figure 5A). Pathway analysis of the 194 common regulated genes revealed the most significant enrichment in several core signaling pathways (Figure 5B). We firstly confirmed the fidelities of microarray results by analyzing the mRNA expression of nine most down-regulated genes with qRT-PCR (Figure 5C), which were tightly associated with cancer stem cell stemness or glioma tumorigenesis. Data of qRT-PCR showed that expression levels of these genes were found to be consistent with that in the microarray data in U251 cells and U87 cells (Figure 5D and 5E). Among these genes, we chose PDK1 as the target downstream of SOX9, which exhibited the most change in SOX9 knockdown cells. Protein expression of PDK1 was detected in four GBM tissues and the couple normal brain tissues. Result showed that PDK1 expressed significant higher in GBM tissues than normal brain tissues (Figure 5F and 5G). Then, the PDK1 expression level was also detected in U251 and U87 GBM cell lines and HEB, and the result showed that the level of PDK1 was higher in both U251 cells and U87 cells than in HEB cells (Figure 5H and 5I). Western blot analysis also indicated that PDK1 was significantly decreased in U251 (Figure 5J and 5K) and U87 (Figure 5L and 5M) cells. In a summarization, these results underlined that PDK1 was a downstream target of SOX9.

### Inactivation of PDK1 inhibited glioma cell colony formation and sphere formation

**Figure 6:**
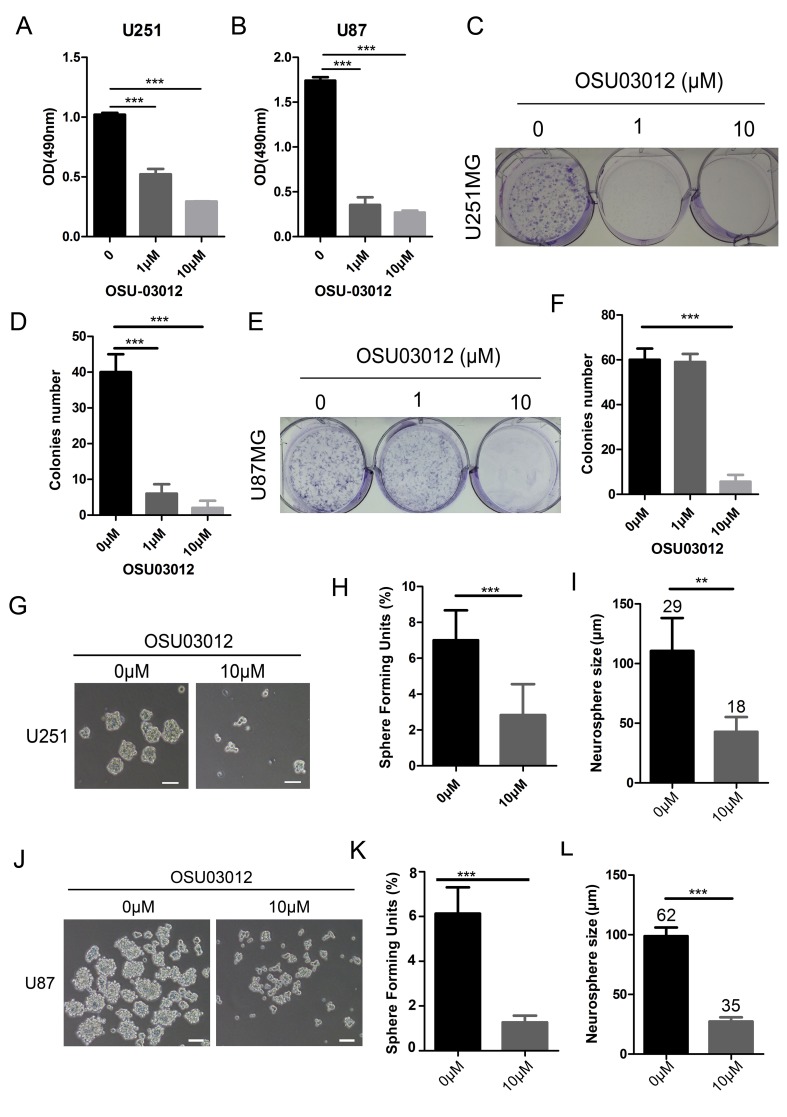
Inactivation of PDK1 inhibited glioma cell colony formation and glioma sphere formation **(A)** Cell viability of U251 cells exposing in OSU03012. (**B)** Cell viability of U87 cells exposing in OSU03012. (**C)** The morphology of U251 cell colonies formed by OSU03012. (**D)** Number of cell colonies formed by OSU03012 in U251 cells. (**E)** The morphology of U87 cell colonies formed by OSU03 012. (**F)** Number of cell colonies formed by OSU03012 in U87 cells. (**G)** The morphology of tumor spheres formed by the glioma stem-like cells from control U251 and OSU03012 in U251 cells. (**H)** Effect of OSU03012 on the number of Sphere Forming Units (SFU) of U251 cells. (**I)** Effect of OSU03012 on the size of U251 GSCs, Arabic numerals represented for spheres number. (**J)** The morphology of tumor spheres formed by the glioma stem-like cells from control U87 and OSU03012 U87 cells. (**K)** Effect of OSU03012 on the number of Sphere Forming Units (SFU) of U87 cells (**L)** Effect of OSU03012 on the size of U87 GSCs, Arabic numerals represented for spheres number. Error bars represent SD. The densitometry data were expressed as the mean ± SD of three independent experiments. ^*^P < 0.05, ^**^P < 0.01, ^***^P < 0.001 versus non-treated cells.

On account of that PDK1 is a normal upstream of AKT pathway, and AKT pathway is an important regulator in cancer stem cell. So we speculated that PDK1 might be a potential target in SOX9 stemness regulating. To detect the role of PDK1 in glioma stemness, the activity of PDK1 was inhibited by a PDK1-specific inhibitor OSU03012. First, we verified that the protein expression of PDK1 was decreased in SOX9-knockdown U251MG (Figure 5E) and U87MG cells (Figure 5G). Then we found that cell viabilities of U251MG and U87MG were decreased by OSU03012 (Figure 6A and 6B). Then colony formation and sphere formation assay were performed to see the effect of OSU03012 in glioma cell growth and glioma stemness. Data showed that the colony formation capacities was significant decreased in both in U251MG (Figure 6C and 6D) and U87MG (Figure 6E and 6F) when exposing in OSU03012 compared with the negative controls. While the PDK1 activator PS48 didn’t reveal effect to glioma cell colony formation (Supplementary Figure 1A–1D). Sphere formation assay showed that OSU03012 could effectively inhibit sphere formation of U251MG (Figure 6G–6I) and U87MG (Figure 6J–6L) cell lines. Meanwhile, the mean diameter of the spheres was smaller when exposing with OSU03012. Interestingly, PS48 also had no influence to the sphere formation capacity to glioma cells (Supplementary Figure 2A–2E).

### PDK1 inactivity sensitized GSC to TMZ treatment both *in vitro* and *in vivo*

**Figure 7:**
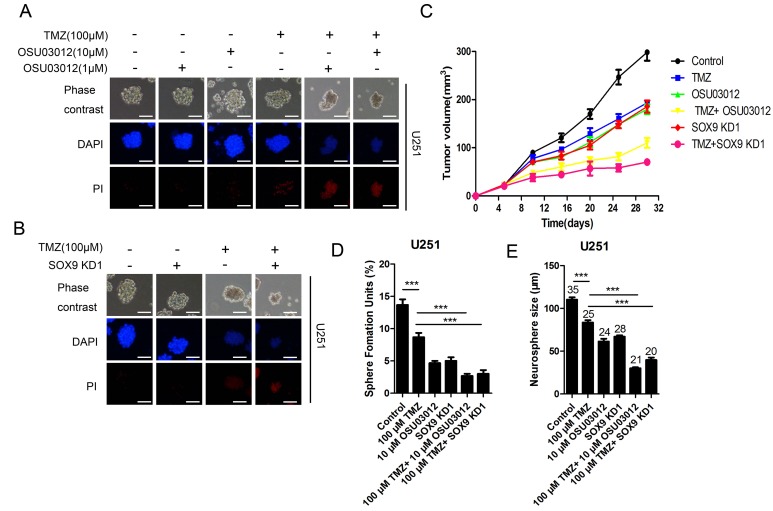
PDK1 and SOX9 knockdown sensitized GSC to TMZ *in vitro* and *in vivo* **(A)** PI/Hoechst assay of U251MG sphere treated with TMZ and OSU03012. (**B)** PI/Hoechst assay of U251MG-SOX9 KD1 sphere treated with TMZ. (**C)** Growth curves of different groups of nude mice xenograft tumor. (**D)** Number of Sphere Forming Units (SFU) of U251 GSC with different treatment. (**E)** The size of U251 GSC with different treatment, Arabic numerals represented for spheres number.

To determine whether PDK1 activity was related with temozolomide (TMZ) resistance of glioma stem cell, glioma GSCs were incubated with serial dilutions of TMZ with or without PDK1 inhibitor OSU03012 and PDK1 activator PS48, then GSC spheres number and size were evaluated. The results showed that the spheres number of GSC treated with TMZ and OSU03012 was obviously decreased that only treated with TMZ both in U251-GSC (Figure 7D and Supplementary Figure 3E) and U87-GSC (Figure 7E and Supplementary Figure 3F). The effect of PDK1 activity on GSC cell apoptosis was investigated by PI/Hoechst staining. The treatment combined of 100 μmol/L TMZ and 1 μmol/L or 10 μmol/L OSU03012 for 12 h induced substantial apoptosis in U251MG (Figure 7A) and U87MG (Supplementary Figure 3) GSCs, while 100 μmol/L TMZ alone only caused a very slight cell apoptosis. The effect of TMZ on GSC apoptosis was consistent with SOX9-shRNAs, data showed that there were apparent more PI positive cell in GSC that co-treated with 100 μmol/L TMZ and SOX9 shRNA1 for 12 h than GSC that single treated with TMZ or SOX9 (Figure 7B and Supplementary Figure 3B). OSU03012 alone could also induce U251MG and U87MG GSC apoptosis (Supplementary Figure 3C and 3D), but the treatment time must be more than 48 h, which was much longer than combination of TMZ and OSU03012. Moreover, the combination of PS48 and TMZ didn’t induce cell apoptosis increase U87MG in GSCs (Supplementary Figure 4), indicating that PDK1 activation could enhance GSC resistance to TMZ. Finally, the effect of PDK1 activity or SOX9 expression level on GSC was assessed *in vivo* using nude mice. The result showed that the tumor treated by TMZ combined with SOX9 shRNA or PDK1 inhibitor grew much slower that TMZ single, indicating that SOX9 shRNA and PDK1 inhibitor could significant enhance the suppressing effect of TMZ on nude mice tumor growth (Figure 7C). Taken together, PDK1 inactivity sensitized GSC to TMZ treatment both *in vitro* and *in vivo.*

**Figure 8:**
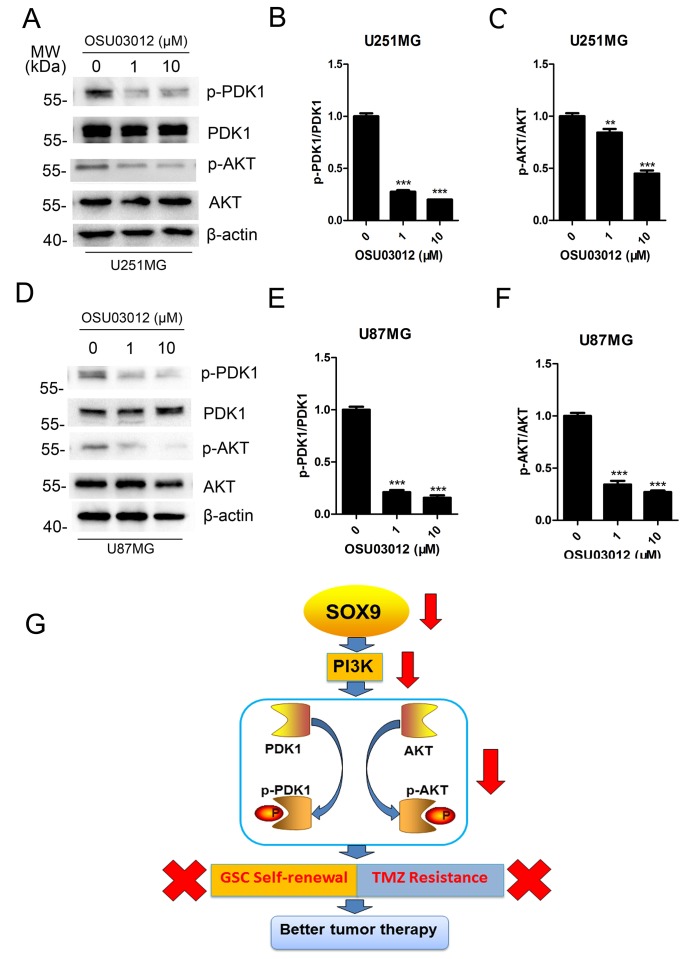
Mechanism of SOX9-PDK1 regulation axis in GSC self-renewal and TMZ resistance **(A)** Western blots analysis for p-PDK1, PDK1, p-AKT and AKT expression in U251MG after exposing in OSU03012 for 4 h. (**B–C)** Quantitation of p-PDK1/PDK1 and p-AKT/AKT in U251MG. (**D)** Western blot analysis for p-PDK1, PDK1, p-AKT and AKT expression in U87MG after exposing in OSU03012 for 4 h. (**E–F)** Quantitation of p-PDK1/PDK1 and p-AKT/AKT in U87 MG. (**G**) Pattern diagram of SOX9-PDK1 axis in GSC self-renewal and TMZ resistance. The densitometry data were expressed as the mean ± SD of three independent experiments. ^**^P < 0.01, ^***^P < 0.001 versus non-treated cells.

In human glioma, PI3K-AKT signaling promotes cell growth, cell survival and glioma stem cell property by several mechanisms, and AKT pathway is reported to a key axis in glioma stemness and tumorigenesis [20]. So we detected the change of AKT to determine whether OSU03012 induced GSC apoptosis by regulate AKT pathway. Western blotting analysis showed that the expression of p-PDK1 and p-AKT in GSCs were down-regulated after disposed in OSU03012 both in U251MG (Figure 8A–8C) and U87MG (Figure 8D–8F), indicating that the inhibition of AKT pathway was the regulator for PDK1 facilitating TMZ sensitization. Together, the above results indicated that SOX9-PDK1 axis was a key regulator for glioma stem cell properties which were PI3K-AKT pathway dependently.

## DISCUSSION

SOX9 belongs to group E (*SOX8*, *SOX9*, and *SOX10*) of the SOX transcription factor family defined by a common HMG box domain originally identified in SRY, the sex-determining gene on the Y chromosome [21]. SOX9 has roles in epithelial invasion, migration, and proliferation and plays important roles in multiple types of cancers [22–24]. What’s interest is that SOX9 is regulated by many upstream pathway including EGFR, Notch, Wnt and sonic hedgehog pathways [25–28]. These pathways were reported to be associated with CSC regulation. In GBM, SOX9 protein expression suppressing in the glioma cell lines was reported displayed loss of cell adhesion, inhibition of AKT phosphorylation and G1 arrest [29]. These studies prompt SOX9 may be a critical regulator in glioma tumorigenesis.

PI3Ks are a family of heterodimeric lipid kinases composed of catalytic and regulatory subunits that, on stimulation, catalyze production of the second messenger phosphatidylinositol-3,4,5-triphosphate (PIP3). Recent studies have reported that PI3K-AKT pathways can be activated by SOX9. SOX9 directly binds to the promoter region of the PI3K subunit gene Pik3ca (also known as p110, one of three subunit proteins of PI3K), enhancing the phosphorylation of AKT [30]. PI3K generates phosphatidylinositol (3,4,5)-trisphosphate [PtdIns(3,4,5)P3] from PtdIns(4,5)P2. PtdIns(3,4,5)P3 causes phosphorylation of and activates AKT [31]. PDK1 is also a downstream kinase of PI3K, and it can be activated by PI3K. PIP3 brings PDK1 and AKT to the membrane, where PDK1 activates AKT by phosphorylation at residue threonine 308 [32, 33]. In human glioma, PI3K-AKT signaling promotes cell growth, cell survival and glioma stem cell property by several mechanisms [34]. But the role of SOX9-PDK1 signaling in glioma tumorigenesis and glioma stem cell has not been reported.

In this paper, SOX9 was demonstrated to be a key regulator in glioma tumorigenesis and GSC self-renewal. For a deep exploration of the downstream gene spectrum of SOX9, we screened through Gene Expression Chip to find the list of differentially expressed genes regulated by SOX9. PDK1 is indicated the downstream target of SOX9 that showed similar function with SOX9 in GSC self-renewal and TMZ resistance. Our data showed that SOX9 and PDK1 exhibited the similar function in glioma colony formation and sphere formation. What’s more, SOX9 knockdown could remarkably decrease the level of p-PDK1 and p-AKT, while PDK1 inactivation could also decrease the level of p-AKT. Due to the important role of PI3K/AKT in both cancer progression and stem cell self-renewal [29, 30], it is reasonable that SOX9-PDK1 axis might be very important in glioma stemness properties. Meanwhile, our data proved that SOX9 knockdown and PDK1 inactivation both greatly decreased GSC stemness and sensitized GSC to TMZ treatment. Thus, we constructed the theory that SOX9-PDK1 axis regulated GSC self-renewal and GSC TMZ resistance by regulating the activation of PI3K-AKT pathway (Figure 8G). This is the first time that SOX9-PDK1 axis is indicated as a key regulator in glioma stem cell maintenance. Considering the versatile functions of SOX9 in cancer biology, further research such as expression profile and proteomic analysis will be conducted to explore the entire mechanism framework of SOX9 on the regulation of cancer stemness in the future.

In a conclusion, our work provides a new insight into the maintenance mechanism of glioma stemness by uncovering the novel SOX9-PDK1 axis, and intervention of which will be a promising alternative approach for the radical cure of GBM.

## MATERIALS AND METHODS

### Human GBM tissue preparation

Human GBM samples were obtained from surgery operations in Tangdu Hospital in Xi’an. The normal brain tissues were obtained from the patients of traumatic brain edema that underwent partial brain resection, which were preserved in liquid nitrogen. All procedures related to acquiring patient samples were approved by the ethics committee of Tangdu Hospital Institutional Review Board. The tissues were broken by homogenizer, then total mRNA and protein were extracted.

### Cell lines and sphere formation assay

The HEB, U251MG and U87MG cell lines were purchased from the Chinese Academy of Sciences Cell Bank [18]. The authenticity of cancer cell lines was tested by short tandem repeat profiling. All cell lines were grown in Dulbecco’s Modified Eagle Medium (DMEM) supplemented with 10% FBS (Gibco, USA). Glioma stem-like cells (GSCs) were cultured and isolated from U251 and U87MG glioma cell lines by using serum-free medium (SFM) which was supplemented with 20 ng/mL basic fibroblast growth factor (bFGF; Sigma-Aldrich, USA), 20 μL/mL B27 supplement (Life Technologies), and 20 ng/mL EGF (Sigma-Aldrich, USA). The medium was refreshed every 2 days. After 10 days, the tumor spheres diameters larger than 50 μm were counted and photographed using phase contrast microscope [19]. The number of Sphere Forming Units (SFU) is calculated according to the following formula: SFU = (number of spheres counted/number of input cells) × 100.

### Lentivirus transfection and colony formation assay

Stable shRNA-expressing U251 and U87MG cell lines were established by puromycin screening at a concentration of 2 μg/mL for about two weeks to screen stably transfected U251 and U87MG cells with SOX9 shRNA and control shRNA. The target sequences are NC: TTCTCCGAACGTGTCACGT; SOX9 KD1: GCATCCTTCAATTTCTGTATA; SOX9 KD1: CTCCACCTTCACCTACATGAA. For clonogenicity, 1 × 10^3^ cells were seeded in 60 mm petri dish. After about 7 to 10 days culturing, colonies were dyed by crystal violet and the colonies number was calculated and collected for statistical analysis.

### Quantitative RT-PCR

Total RNA from glioma cells was isolated using TRIzol reagent (Invitrogen, USA). The RNA was subsequently treated with RNase-free DNase I (Roche, Switzerland). Synthesis of cDNA was done by using the BcaBest RNA PCR kit from TaKaRa (Japan) according to the manufacturer’s instructions. Quantitative RT-PCR was carried out using the iQ5 Multicolor Real-Time PCR Detection System (Bio-Rad) with Realtime PCR Master Mix (SYBR Green). The PCR primers are listed in Supplementary Table 1, β-actin was selected as the endogenous control in the assay.

### Western blotting

The total cell lysates were prepared in RIPA lysis buffer with complete protease inhibitor cocktail (Beyotime, China). The protein concentration was determined using a BCA Protein Assay Kit (Beyotime, China). The cell lysates were added to SDS sample buﬀer (BioRad), boiled for 5 minutes, and separated using SDS-PAGE. The proteins were transferred onto nitrocellulose membranes, which were subsequently blocked in 5% milk. The membranes were incubated with primary antibodies according to the manufacturers’ protocols. The dilutions of primary antibodies in Western blot assay were SOX9 (1:4000, Abcam, UK), β-actin (1:4000, Abmart, China), p-AKT (1:1000, Cell Signaling Technology, USA), AKT (1:1000, Cell Signaling Technology, USA), p-PDK1 (1:1000, Cell Signaling Technology, USA), PDK1 (1:1000, Cell Signaling Technology, USA), NESTIN (1:2000, Sigma-Aldrich, USA), SOX2 (1:1000, Sigma-Aldrich, USA), respectively. The membranes were incubated with horseradish peroxidase (HRP)-conjugated secondary antibodies for 1 hour at room temperature. The immunoblots were developed using Amersham™ ECL (GE Healthcare), and images were acquired using the luminescent image analyzer LAS-4000 mini (Fujiflm, GE Healthcare). The western blot signal representing protein level was obtained by quantifcation using ImageJ software.

### Temozolomide sensitive cell death assay

Glioma spheres grown in the presence of serum were seeded in 24-well plates. Spheres were left untreated or treated with TMZ (Tasly Pharmaceutical Co., Ltd., Tianjin, China). Then spheres were incubated in culture medium containing 5 mg/mL PI and 5 mg/mL Hoechst at 37°C for 30 min. Cell death was observed under fluorescence microscope, and cell death ratio was quantified by the ratio of PI/ Hoechst.

### Microarray analyses

Total RNA was extracted using TRIzol reagent (Invitrogen, USA). Extracted RNA was labeled and hybridized onto the Agilent Human Gene Expression Analysis platform (8*60K, Design ID: 039494) by Oebiotech Co.. Statistical analyses and data normalization were conducted using the Genespring GX software (Agilent Technologies, USA). Genes with 1.5-fold change in expression were considered differentially regulated by SOX9. Genes were mapped onto KEGG pathways using DAVID version 6.7 (http://david.abcc.ncifcrf.gov/).

### Tumor xenografts assay

To test the anti-glioma effect of TMZ alone or in combination with SOX9 shRNA and PDK1 inhibitor OSU03012 *in vivo*, a xenograft model of human glioma was established. 4-week old male SCID mice were purchased from Vital River Laboratory Animal Technology Co. Ltd. (Beijing, China). Each mouse was injected subcutaneously in the right flank with 1 × 10^6^ U87 GSCs resuspended in 200 μL PBS. The mice of SOX9 KD group and TMZ combined SOX9 KD group were injected with SOX9-shRNA1 stable expressing U87 GSCs, and the other groups were injected with normal U87 GSCs. Mice were randomly divided into 6 groups (five mice per group) and treatment was started. The TMZ treated concentration is 25 mg/kg/day, and the OSU03012 treated concentration is 15 mg/kg/day. Tumor diameter was measured every 2 days, and the tumor volume was calculated (length × width × width × 0.5).

### Statistical analysis

Statistical differences were analyzed using Student’s *t*-test for unpaired samples. An ANOVA followed by the Dunnett’s test was used for multiple comparisons with one control group. The criterion for significance (*p* value) was set as mentioned in the Figure legends.

## SUPPLEMENTARY MATERIALS FIGURES AND TABLE


