# Supplementary material for: SOX9-PDK1 axis is essential for glioma stem cell self-renewal and temozolomide resistance
Source: Oncotarget. 2017 Nov 30;9(1):192–204. doi: 10.18632/oncotarget.22773 (PMC5787456; doi:10.18632/oncotarget.22773)
Supplement: Supplementary file 1 [file oncotarget-09-192-s001.pdf]

SOX9-PDK1 axis is essential for glioma stem cell self-renewal and temozolomide resistance

SUPPLEMENTARY MATERIALS

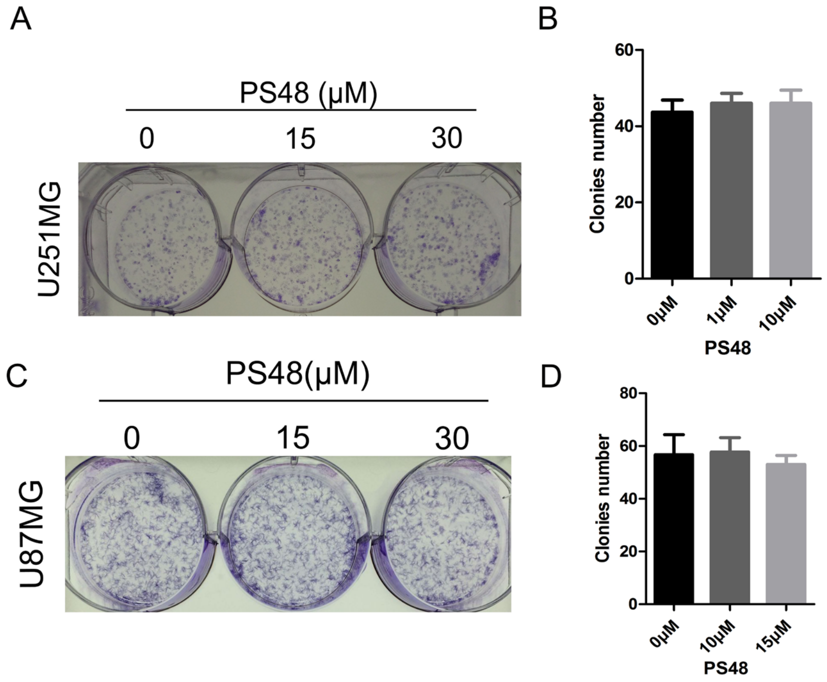

Supplementary Figure 1: Effect of PS48 on glioma colony formation. (**A**) The morphology of cell colonies formed by U251 cells exposed with PS48. (**B**) Number of cell colonies formed by U251 cells exposed with PS48. (**C**) The morphology of cell colonies formed by U87 cells exposed with PS48. (**D**) Number of cell colonies formed by U87 cells exposed with PS48.

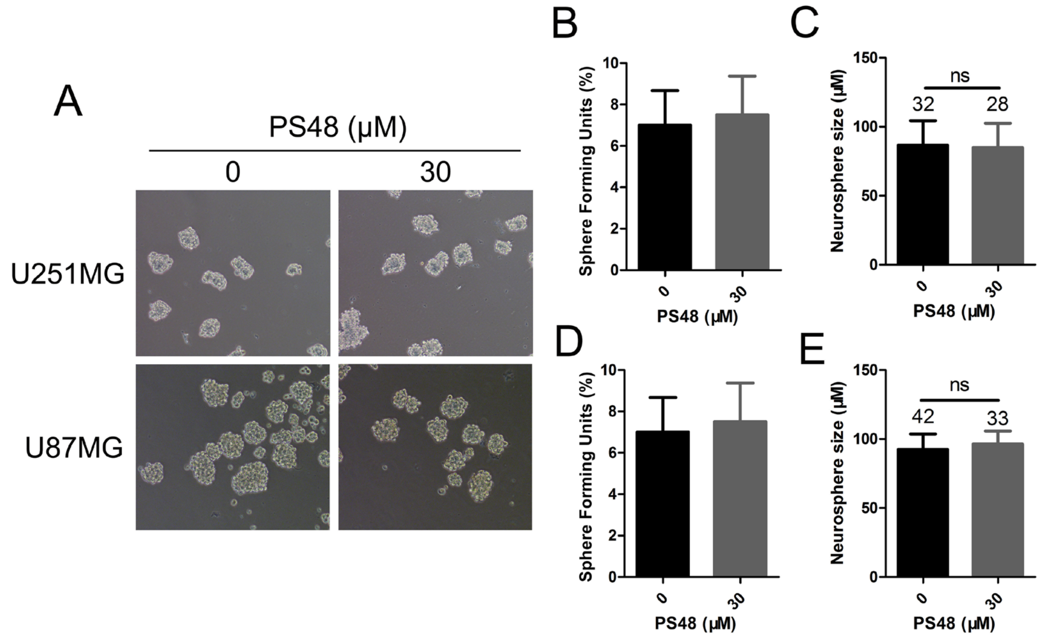

Supplementary Figure 2: Effect of PS48 on glioma cell sphere formation. (**A**) Represented sphere images of U251MG and U87MG. (**B**) SFU of U251MG cells exposed with PS48. (**C**) Diameters of U251MG spheres, Arabic numerals represented for spheres number. (**D**) SFU of U87MG cells exposed with PS48. (**E**) Diameters of U87MG spheres, Arabic numerals represented for spheres number.

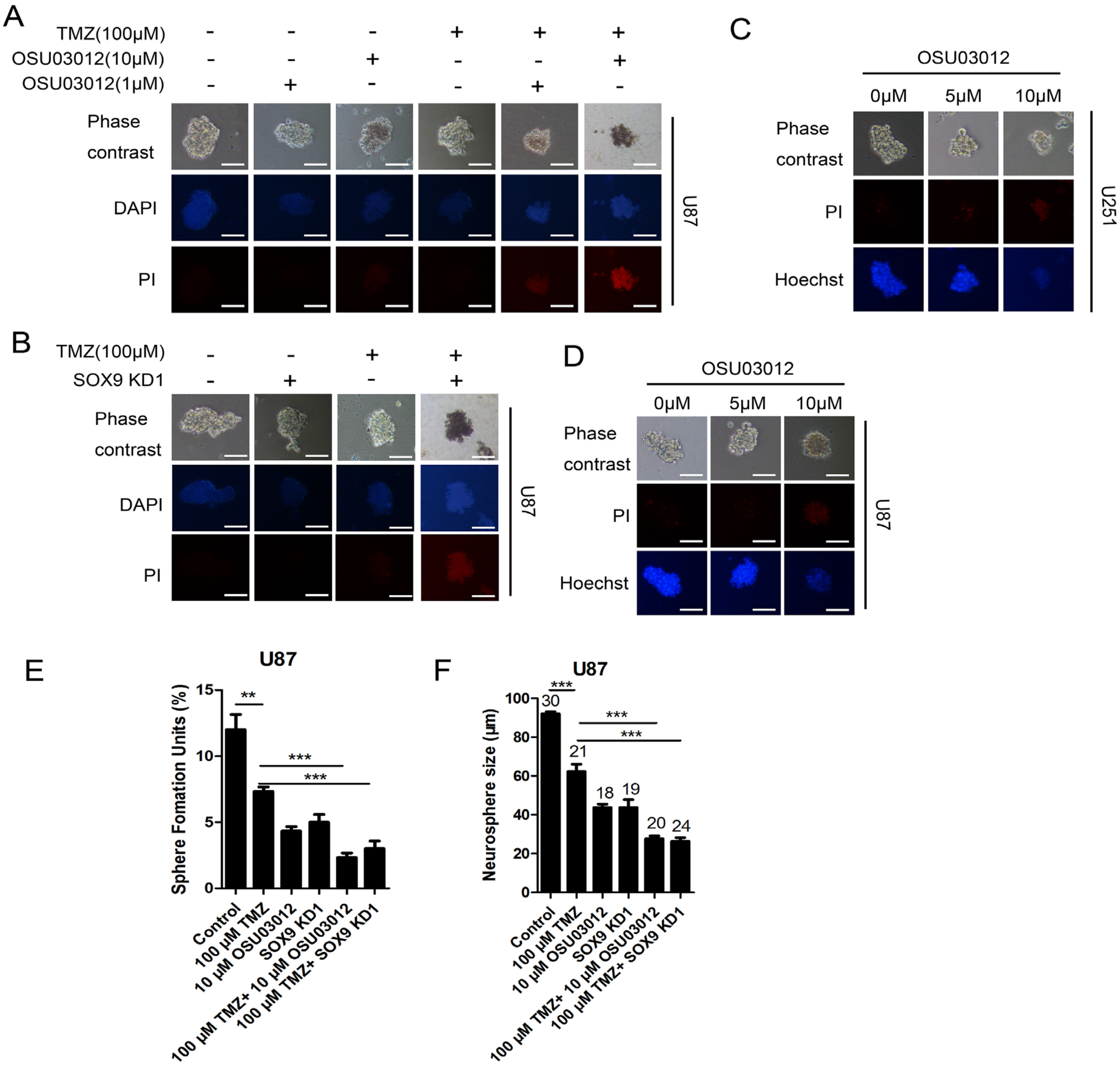

Supplementary Figure 3: PI/Hoechst assay for U87MG sphere with different combination of TMZ and OSU03012. (**A**) PI/Hoechst assay of U251MG sphere treated with TMZ and OSU03012 for 12 h. (**B**) PI/Hoechst assay of U251MG-SOX9 KD1 sphere treated with TMZ for 12 h. (**C**) PI/Hoechst assay of U251MG sphere treated with OSU03012 for 48 h. (**D**) PI/Hoechst assay of U87MG sphere treated with OSU03012 for 48 h. (**E**) Number of Sphere Forming Units (SFU) of U87 GSC with different treatment. (**F**) The size of U87 GSC with different treatment, Arabic numerals represented for spheres number.

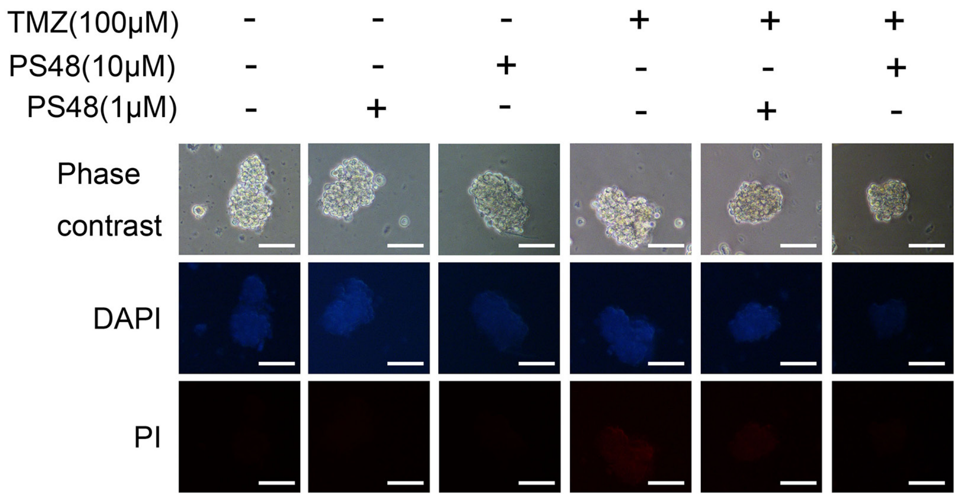

Supplementary Figure 4: PI/Hoechst assay for U87MG spheres with different combination of TMZ and PS48.

Supplementary Table 1: Primer sequences used in the study via real-time PCR analysis

Gene	Oligonucleotide	Primer sequences
PDFGRA	Upper primer	5′- GACGAGACCATCGAGGACAT -3′
	Lower primer	5′- GCCTCGGGAACTTTCTCTCT -3′
ARHGAP29	Upper primer	5′- CTCCCAAGTAGAGGCTGCAC -3′
	Lower primer	5′- GCTGGTGAATGATGTCAGGA -3′
IGFBP5	Upper primer	5′ - AGAGACTCTCGGGAGCATGA -3′
	Lower primer	5′- ATCTCAGGTGCAGGGATGAC -3′
PTPRZ1	Upper primer	5′-TCCTATACAGGAGCACTGAATCA-3′
	Lower primer	5′-TGACACGGTAGTCATTAGTGAGA-3′
TET1	Upper primer	5′- TCTTCCCCATGACCACATCT -3′
	Lower primer	5′- GAGGGAAAAGAAGCCCAAAG -3′
CD44	Upper primer	5′ - TCCAACACCTCCCAGTATGACA -3′
	Lower primer	5′ - GGCAGGTCTGTGACTGATGACA -3′
NDRG1	Upper primer	5′- CTCTGTTCACGTCACGCTGT -3′
	Lower primer	5′- AGAGGGGGTTGTAGCAGGTT -3′
SOX9	Upper primer	5′- AGGTGCTCAAAGGCTACGACT-3′
	Lower primer	5′- AGATGTGCGTCTGCTCCGTG -3′
Nestin	Upper primer	5′- AGCAGGAGGAGTTGGGTTCT-3′
	Lower primer	5′- AGTGGAGTCTGGAAGGGTCTC-3′
SOX2	Upper primer	5′- ATGGGTTCGGTGGTCAAGT-3′
	Lower primer	5′- GCTCTGGTAGTGCTGGGACA-3′
Nanog	Upper primer	5′-GCAAATGTCTTCTGCTGAGATGC-3′
	Lower primer	5′- GCTGTCCTGAATAAGCAGATCCAT-3′
Oct-4	Upper primer	5′- GTGTTCAGCCAAAAGACCATCT-3′
	Lower primer	5′- GGCCTGCATGAGGGTTTCT-3′
CD133	Upper primer	5′- ACCAGGTAAGAACCCGGATCAA-3′
	Lower primer	5′- CAAGAATTCCGCCTCCTAGCACT-3′
GAPDH	Upper primer	5′- GCACCGTCAAGGCTGAGAAC-3′
	Lower primer	5′- TGGTGAAGACGCCAGTGGA-3′
